# Supplementary material for: Meta-analysis of the brain transcriptomes of multiple genetic mouse models of schizophrenia highlights dysregulation in striatum and thalamus
Source: Transl Psychiatry. 2025 Sep 29;15:345. doi: 10.1038/s41398-025-03563-5 (PMC12480767; doi:10.1038/s41398-025-03563-5)
Supplement: Supplementary file 1 — Supplemental Figures [file 41398_2025_3563_MOESM1_ESM.pdf]

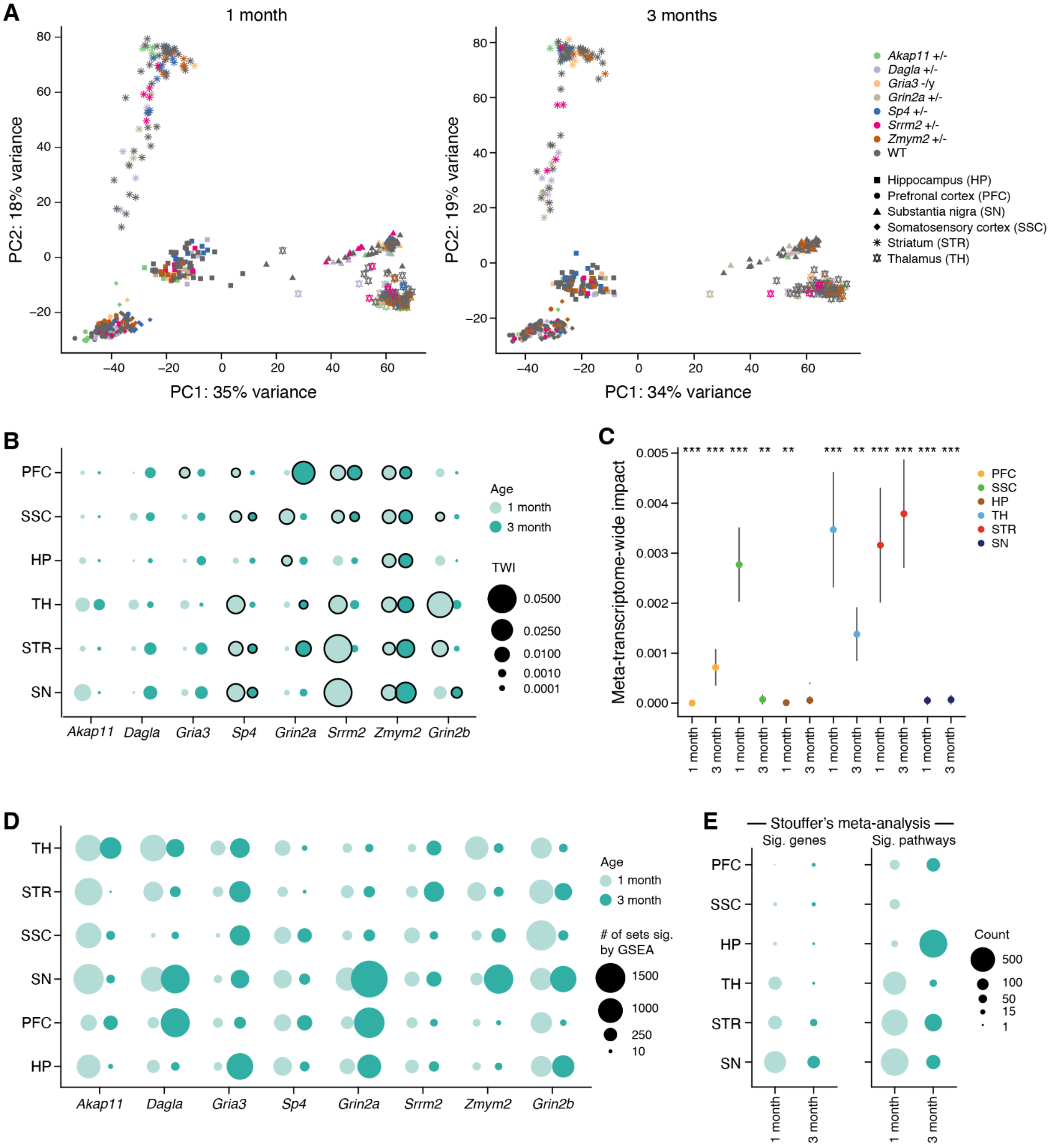

Supplemental Figure 1. Breadth of RNA-sequencing and transcriptomic impact of SCHEMA mutants

A) PCA of all samples included in this meta-analysis, with color representing genotype and shape representing brain region **B)** Transcriptome-wide impact scores for each mutant, brain region, and age, as indicated. Bubble size indicates TWI score, color indicates age, and a black ring indicates nominal significance ($p < 0.05$). **C)** Meta-TWI as meta-analyzed across SCHEMA mutants for each age and brain region. Stars indicate significance, * : FDR < 0.05, ** : FDR < 0.01, *** : FDR < 0.001 **D)** Number of significant gene sets by GSEA for each mutant, brain region, and age. **E)** Number of significant genes and GO gene sets (pathways) by Stouffer's meta-analysis across SCHEMA mutants for each brain region and age.

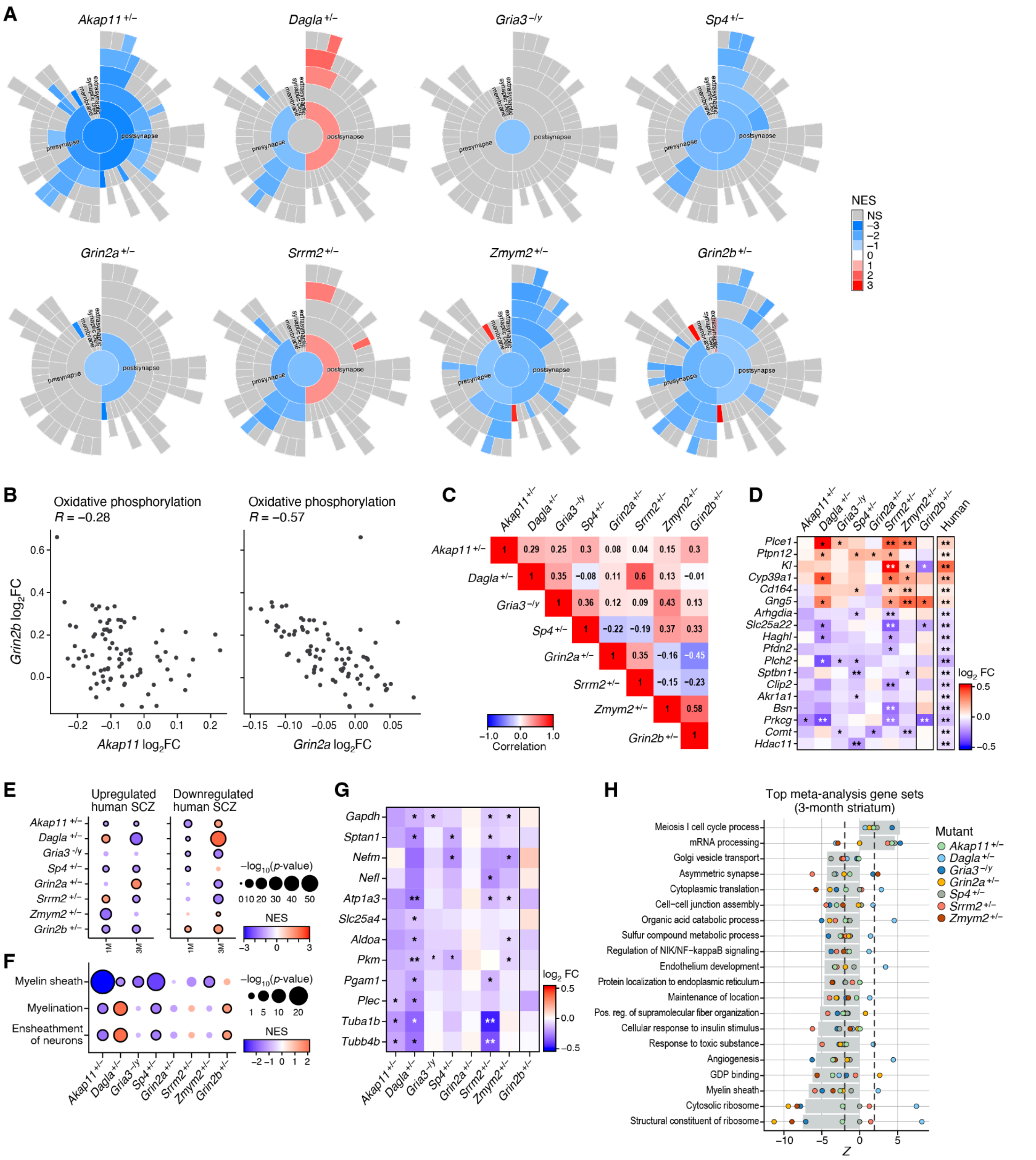

Supplemental Figure 2. Convergence of pathway-level transcriptomic changes in SCHEMA mouse striatum

A) SynGO sunburst plots for GSEA in 1 month striatum. Color represents NES, and non significant terms (FDR > 0.05) are plotted in gray. See Supplemental Figure 5 for extended plot

annotation, and full key to the sunburst plot can be found at the synGO portal https://www.syngoportal.org/ **B)** Correlation of log_2FC for all genes in the “oxidative phosphorylation” GO gene set between SCHEMA mutants (Grin2a and Akap11) and Grin2b **C)** Correlation of GSEA results across mutants. Spearman’s correlation was performed on the union of nominally significant gene sets for each pair of mutants in 1 month striatum. Number represents Spearman’s correlation coefficient. **D)** Genes which are significant and direction-matching by meta-analysis of SCHEMA mutants and in human patient post-mortem striatum * : $p < 0.05$, ** : FDR < 0.05 **E)** GSEA for upregulated DEGs and downregulated DEGs identified in human patient postmortem striatum. Black ring indicates FDR < 0.05. **F)** Individual GSEA results for myelin related gene sets in 1 month striatum. Black ring indicates FDR < 0.05. **G)** Most consistently downregulated genes in the myelin sheath gene set across SCHEMA mutants in 1 month striatum as determined by a rank-sum approach. * : $p < 0.05$, ** : FDR < 0.05. **H)** Top 20 most significant gene set changes by meta-analysis in 3 month striatum across SCHEMA mutants. Gray bar represents Stouffer’s meta Z score, each point represents the individual gene set Z scores from each SCHEMA mutants. Dashed lines correspond to the Z score which represents nominal significance ($p < 0.05$). For the purpose of display, redundant gene sets were collapsed and only the strongest of the similar gene sets is plotted (see Methods)

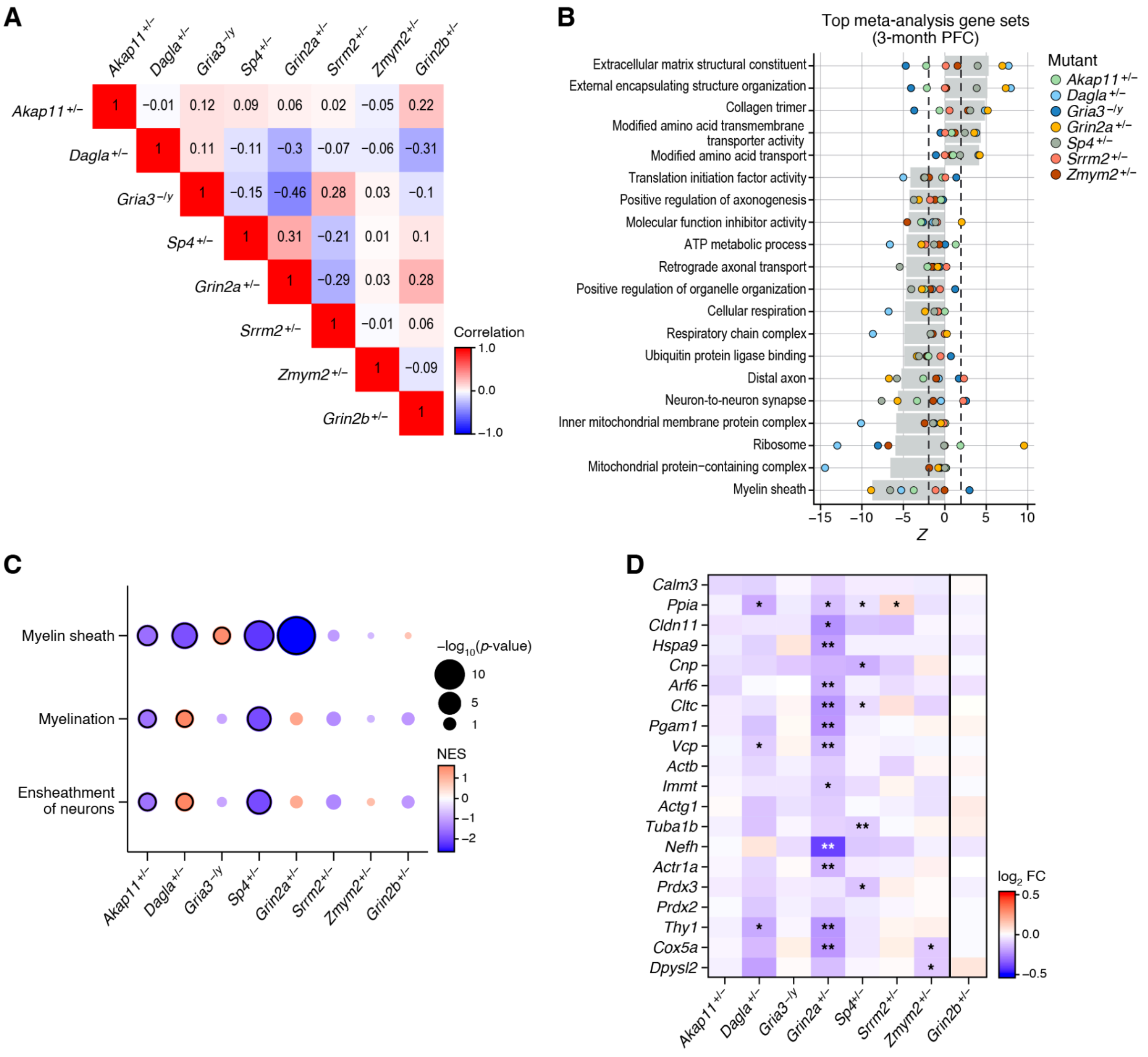

Supplemental Figure 3. Heterogeneity of transcriptomic change in SCHEMA mouse PFC
A) Correlation of DE results in PFC across mutants. Spearman's correlation was performed on the union of nominally significant genes for each pair of mutants in 1 month PFC. Number represents Spearman's correlation coefficient. **B)** Top 20 most significant gene set changes by meta-analysis in 3 month PFC across SCHEMA mutants. Gray bar represents Stouffer's meta Z score, each point represents the individual gene set Z scores from each SCHEMA mutants. Dashed lines correspond to the Z score which represents nominal significance ($p < 0.05$). For the purpose of display, redundant gene sets were collapsed and only the strongest of the similar gene sets is plotted (see Methods) **C)** Individual GSEA results for myelin related gene sets in 3 month PFC. Black ring indicates FDR < 0.05. **D)** Most consistently downregulated genes in the myelin sheath gene set across SCHEMA mutants in 3 month PFC as determined by a rank-sum approach. * : $p < 0.05$, ** : FDR < 0.05

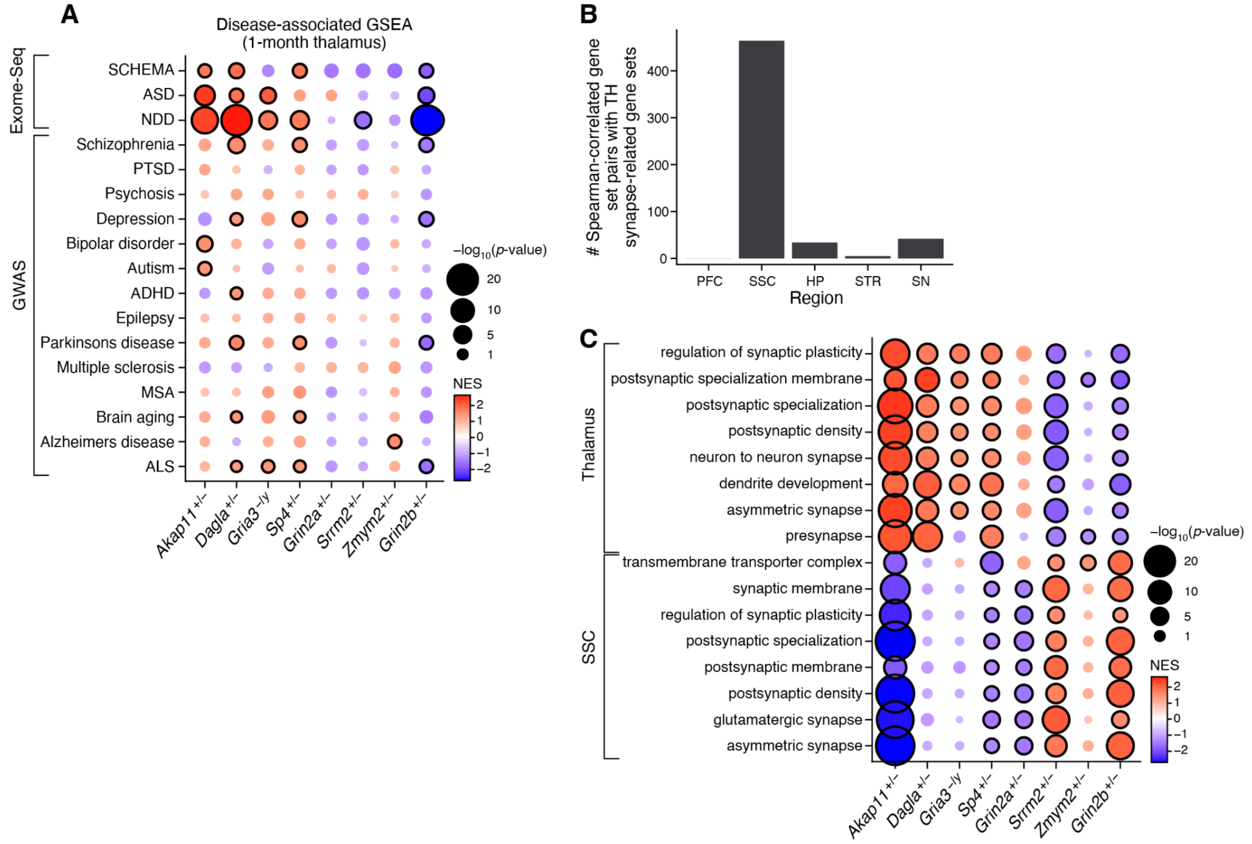

Supplemental Figure 4. Divergence between DD/ID- and non-DD/ID associated SCHEMA mutants in synaptic disease-related pathways

A) GSEA results in 1 month thalamus from disease gene sets based on GWAS and exome sequencing studies. Black ring indicates FDR < 0.05. **B)** Number of significant (FDR < 0.05) gene set correlations between the NES of synapse related gene sets in 1 month thalamus and the NES of any gene set in other regions at 1 month across SCHEMA mutants, as determined by Spearman correlation. **C)** GSEA results from gene sets involved in top synapse-related gene set anticorrelations in 1 month thalamus and SSC. Black ring indicates FDR < 0.05.

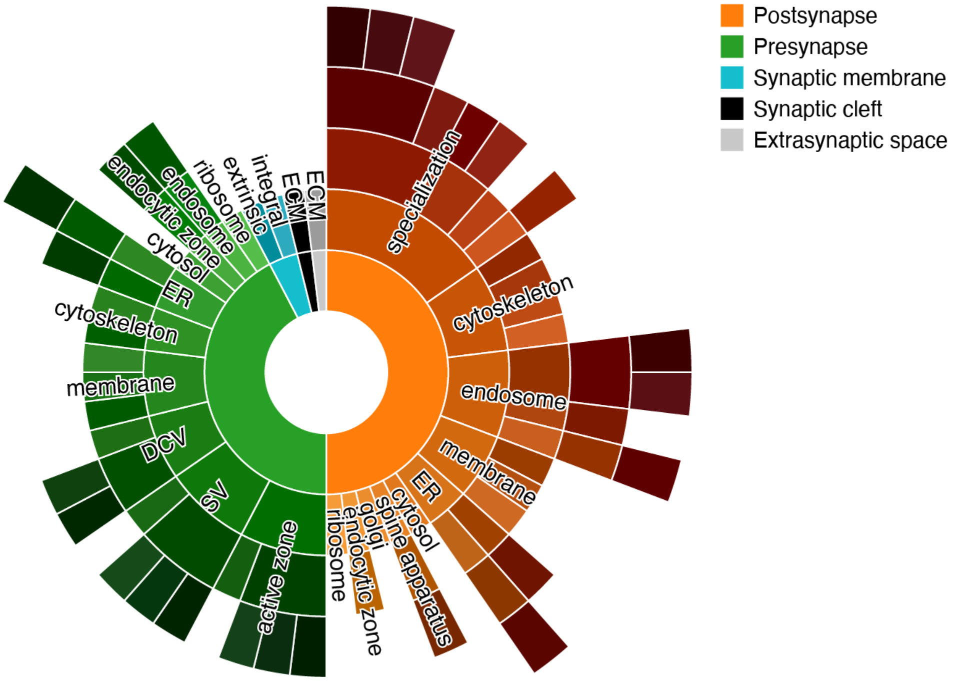

Supplemental Figure 5. Extended annotation of synGO cellular component sunburst plot. Annotation of all plot segments can be found at https://www.syngoportal.org/.

Supplemental Table 1: Samples and QC
Supplemental Table 2: Differential Gene Expression Results
Supplemental Table 3: TRADE results
Supplemental Table 4: Convergence results
Supplemental Table 5: GO Gene Set Enrichment Analysis Results
Supplemental Table 6: GSEA Stouffer's Meta-Analysis Results
Supplemental Table 7: Cell Type Deconvolution Results
Supplemental Table 8: Differential Expression Stouffer's Meta-Analysis Results
Supplemental Table 9: GSEA using DEGs in human SCZ
Supplemental Table 10: synGO GSEA Results
Supplemental Table 11: Disease Gene Set GSEA in 1 month thalamus
Supplemental Table 12: Correlation between 1 month thalamus GSEA and other brain regions
